# Endorsing the STrengthening the Reporting of Observational Studies in Epidemiology-nutritional epidemiology (STROBE-nut) statement at *Genes & Nutrition*

**DOI:** 10.1186/s12263-019-0655-5

**Published:** 2019-11-18

**Authors:** Dana Hawwash, Carl Lachat

**Affiliations:** Department of Food Technology, Safety and Health, Ghent University, Coupure links 653, 9000 Ghent, Belgium

A clear and complete description of study findings is key to ensure that investments in research add maximally to evidence-informed decisions, policies, and interventions. A correct interpretation of study findings and possible bias, however, requires a detailed description of what was done and found. However, many manuscripts may not contain the required information for this purpose [1].

Adequate reporting of research entails a clear and complete description of what was planned, carried out, and found. Over the last decades, reporting guidelines have been developed to assist researchers and increase the completeness of research findings. A reporting guideline is an authoritative statement that includes a checklist of essential items that should be addressed when reporting research findings. The items included in the checklist serve as a guide to authors to include essential information in manuscripts. Reporting guidelines sometimes also contain a flow diagram to visualize the study design [2]. To date, more than 400 reporting guidelines have been developed and are listed on the Enhancing the QUAlity and Transparency of health Research (EQUATOR) Network [3]. Well-known guidelines are the Consolidated Standards Of Reporting Trials (CONSORT), the Preferred Reporting Items for Systematic Reviews and Meta-Analyses (PRISMA) statement, and the STrengthening the Reporting of Observational Studies in Epidemiology (STROBE) statement.

The STrengthening the Reporting of Observational Studies in Epidemiology-nutrition epidemiology (STROBE-nut) statement was developed as a collaborative effort between an international group of nutritionists, epidemiologists, researchers, methodologists, statisticians, and journal editors to improve the reporting in nutritional epidemiology and dietary assessment [4, 5]. As an extension of the STROBE statement, it is applicable to cohort, case-control, and cross-sectional studies. The guidance was developed during a series of face-to-face consortium meetings between 21 experts and three international consultation rounds. STROBE-nut comprises a set of 24 items, organized as a checklist.

An “explanation and elaboration” document was developed to provide detail and justification of each item with examples of clear reporting from published studies [6]. The STROBE-nut checklist is therefore best used together with the STROBE-nut paper, the explanation and elaboration document, and other relevant STROBE extensions, e.g., STROBE-ME for molecular epidemiology [7].

Endorsement of reporting guidelines is key to increase adherence by authors [8]. To date, the STROBE-nut statement is endorsed by various journals including *Genes & Nutrition*, the *International Journal of Behavioral Nutrition and Physical Activity*, *Nutrition Journal*, and the *Journal of the Academy of Nutrition and Dietetics*. With the publication of this article, *Genes & Nutrition* affirms its commitment to the STROBE-nut statement and its goals. In doing so, *Genes & Nutrition* extends its previous recommendations to improve reproducibility and reporting of scientific studies [9].

Guidelines are typically used at the final stage of the writing process, i.e., immediately before submission for publication, driven by a need to respond to journals requirements. Without careful deliberation, stakeholders involved in the publication cycle (i.e. authors, reviewers, editors) might consider reporting guidelines as an administrative encumbrance rather than assistance for authors during write-up. The formative nature of using reporting guidelines, in particular, for scholars and students at early stages of their career has probably not received sufficient consideration. Reporting guidelines are ideally used throughout the writing process to foster familiarity with the recommendations, and good research practices with regard to presentation of research findings.

There are concerns over potential misuse of reporting guidelines. Therefore, we reiterate that reporting guidelines such as STROBE-nut are not a prescription to design or conduct studies. In addition, reporting guidelines should not be used as a quality appraisal tool of studies or as criteria to assess study design. Moreover, the use of reporting guidelines should not restrict the creativity of authors or interfere with the peer review of editorial policies of journals. To ensure correct use and interpretation of STROBE-nut, we therefore recommend that the introduction of reporting guidelines for authors in journals is accompanied by clear instructions regarding correct use for authors, reviewers, and editors.

Appropriate use of STROBE-nut needs the engagement of various stakeholders involved in the research cycle, i.e., from conception of ideas to interpretation of findings. Authors need to be aware of the importance of clear reporting and transparency. Editors can support the use of reporting guidelines by adding them to the instructions for authors, reviewers, and editors. Institutions and funders could develop mechanisms to incentivize adherence to good research practices including the use of reporting guidelines. Higher education institutions could incorporate reporting guidelines in the curriculum of graduate and undergraduate students.

Finally, reporting guideline developers should ensure careful monitoring of the usefulness and appropriateness of recommendations. Regular updates and scrutiny of the timelines and relevance of recommendations and how these are best administered are required [10]. Hence, as part of the consortium that developed STROBE-nut, we welcome contributions and reflections that could improve the use and added value of STROBE-nut. We are particularly interested to learn from researchers about the difficulties faced when using STROBE-nut, i.e., clarity of language, usefulness of the checklist, and comprehensiveness of the recommendations. Such feedback will help to prioritize items during the revisions of the STROBE-nut guidelines.

## Abbreviations

CONSORT: The Consolidated Standards Of Reporting Trials
EQUATOR: The Enhancing the QUAlity and Transparency Of health Research
PRISMA: The Preferred Reporting Items for Systematic Reviews and Meta-Analyses Statement
STROBE: STrengthening the Reporting of Observational Studies in Epidemiology
STROBE-ME: STrengthening the Reporting of Observational Studies in Epidemiology: Molecular Epidemiology
STROBE-nut: STrengthening the Reporting of Observational Studies in Epidemiology-nutritional epidemiology
